# The Roles of Reactive Oxygen Species and Nitric Oxide in Perfluorooctanoic Acid-Induced Developmental Cardiotoxicity and l-Carnitine Mediated Protection

**DOI:** 10.3390/ijms18061229

**Published:** 2017-06-08

**Authors:** Meng Zhao, Qixiao Jiang, Wencheng Wang, Min Geng, Meng Wang, Yantao Han, Chunbo Wang

**Affiliations:** 1 Department of Pharmacology, Qingdao University Medical College, 308 Ning Xia Road, Qingdao 266071, China; zhaom@qdu.edu.cn (M.Z.); 2015020999@qdu.edu.cn (M.G.); 18363991653@163.com (M.W.); hanyt@qdu.edu.cn (Y.H.); 2 Qingdao Municipal Center for Disease Control & Prevention, 175 Shandong Road, Qingdao 266071, China; wwcqdcdc@gmail.com

**Keywords:** perfluorooctanoic acid-induced developmental cardiotoxicity, reactive oxygen species, nitric oxide, l-carnitine, electron spin resonance, p65, iNOS

## Abstract

Perfluorooctanoic acid (PFOA) is an environmental contaminant that could induce developmental cardiotoxicity in a chicken embryo, which may be alleviated by l-carnitine. To explore the roles of reactive oxygen species (ROS) and nitric oxide (NO) in such changes and the potential effects of l-carnitine, fertile chicken eggs were exposed to PFOA via an air cell injection, with or without l-carnitine co-treatment. The ROS and NO levels in chicken embryo hearts were determined with electron spin resonance (ESR), and the protein levels of the nuclear factor κ-light chain-enhancer of activated B cells (NF-κB) p65 and inducible nitric oxide synthase (iNOS) in chicken embryo hearts were assessed with western blotting. The results of ESR indicated that PFOA exposure induced an elevation in the ROS levels in ED19 chicken embryo hearts and hatchling chicken hearts, while l-carnitine could alleviate such changes. Meanwhile, increased NO levels were observed in ED19 embryo hearts and hatchling hearts following PFOA exposure, while l-carnitine co-treatment exerted modulatory effects. Western blotting revealed that p65 translocation in ED19 embryo hearts and hatchling hearts was enhanced by PFOA, while l-carnitine co-treatment alleviated such changes. iNOS expression levels in ED19 embryo hearts followed the same pattern as NO levels, while a suppression of expression was observed in hatchling hearts exposed to PFOA. ROS/NF-κB p65 and iNOS/NO seem to be involved in the late stage (ED19 and post hatch) of PFOA-induced developmental cardiotoxicity in a chicken embryo. l-carnitine could exert anti-oxidant and NO modulatory effects in the developing chicken embryo hearts, which likely contribute to its cardioprotective effects.

## 1. Introduction

Perfluorooctanoic acid (PFOA) belongs to the perfluoroalkyl acids (PFAAs) family, and is widely used in the production of myriad polymer products, such as polytetrafluoroethene (PTFE). These polymers have a wide application in human lives, and act as non-stick coatings, water repellents, and fire retardants [1]. The mass production of PFOA started in the 1950s; however, over the last two decades, PFOA has started to be associated with adverse health outcomes. In highly exposed populations, PFOA exposure has been associated with increased risks of kidney and testicular cancer, pregnancy-induced hypertension, thyroid disease, dyslipidemia, liver damage, rheumatoid arthritis, endocrine disruption, and immunosuppression [2,3,4,5,6,7,8]. In the laboratory, established adverse outcomes of PFOA exposure include immunotoxicity, carcinogenesis, and developmental toxicity [9,10,11]. PFOA-induced developmental cardiotoxicity in a chicken embryo and hatchling chickens was first reported by Jiang et al. [12]. An altered chicken cardiac function and morphology were observed in chicken embryo hearts developmentally exposed to PFOA, which were partially associated with PPAR α and BMP2 signaling [13]. Recently, decreased l-carnitine and its short-chain derivatives levels were also associated with such effects [14]. Considering the ubiquitous presence of PFOA in the environment and biota [15], such effects could be affecting the cardiovascular health of the general population and are worth further investigation.

l-carnitine is an endogenous quaternary ammonium nutrient, which plays an important role in fatty acid metabolism by transporting long chain fatty acids into the mitochondria and short chain acetyl and propionyl groups from the mitochondria to cytoplasm [16]. l-carnitine is known to be cardiac protective against both chemotherapeutic agents [17] and congenital cardiomyopathy [18]. In our previous study [14], developmental exposure to PFOA decreased the l-carnitine, acetyl-l-carnitine, and propionyl-l-carnitine levels in chicken embryo and hatchling chicken hearts, while the extragenous supplement of l-carnitine effectively reverted PFOA-induced developmental cardiotoxicity. l-carnitine definitely plays a role in heart development, and has the potential to serve as a prophylaxis agent to improve public cardiovascular health.

Both PFOA and l-carnitine could affect reactive oxygen species (ROS) generation and nitric oxide (NO) production [19,20]. Elevated oxidative stress could damage cardiomyocytes, alter the heart morphology and function, and deteriorate the prognosis of patients with cardiovascular diseases [21]. PFOA has been reported to elevate ROS and NO levels, which is believed to be part of its mechanism of toxicity [22]. On the other hand, l-carnitine has mixed roles in ROS and NO generation: technically, the presence of l-carnitine could promote fatty acid β-oxidation, which suggests that it may increase ROS generation, as reported in Moraes et al. [23]. However, controversies do exist, as Mescka et al. indicated that l-carnitine supplementation actually decreased the ROS levels in a rat’s brain [24]. A similar controversy also applies for NO, where both an increase and decrease in NO levels were reported following l-carnitine supplementation [25,26]. Such controversies highlight the need for further investigations regarding the effects of l-carnitine on ROS and NO, which may contribute to its cardiac protective effects against PFOA-induced developmental cardiotoxicity.

In this study, ROS and NO levels were directly measured with the electron spin resonance (ESR) technique in chicken embryo hearts developmentally exposed to PFOA/l-carnitine. PFOA exposure remarkably increased the ROS levels in the ED19 chicken embryo and hatchling chicken hearts, while l-carnitine supplementation alleviated such changes. On the other hand, PFOA increased the NO levels in the ED19 and hatchling chicken hearts, while l-carnitine further increased the NO levels in ED19 chicken embryo hearts, but reverted it to a normal level in hatchling chicken hearts. The nuclear factor κ-light chain-enhancer of activated B cells (NF-κB) p65 and inducible nitric oxide synthase (iNOS) has been associated with such changes.

## 2. Results

### 2.1. General Parameters of the Embryonic Day 6 (ED6), 10, 15, and 19 Chicken Embryos and Hatchling Chickens

**Figure 1:**
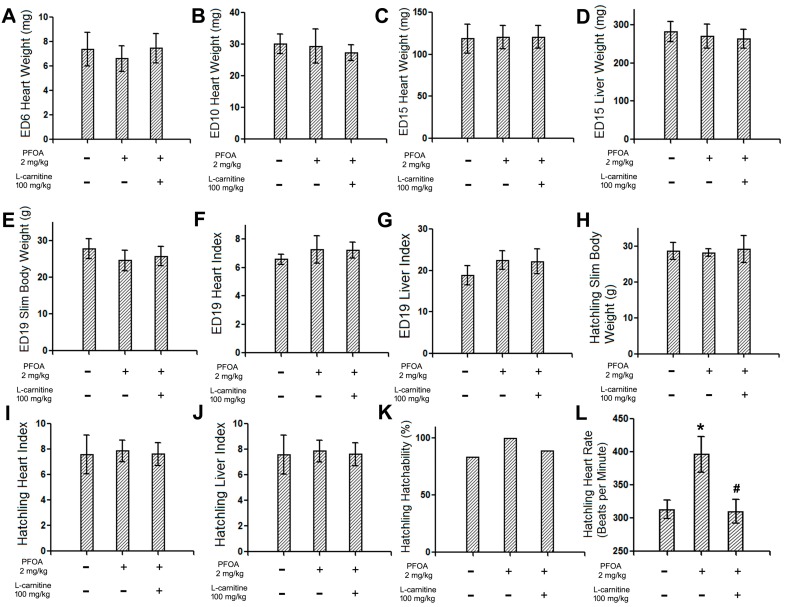
The general parameters of the embryonic day 6 (ED6), 10, 15, and 19 chicken embryos and hatchling chickens and the heart rates of the hatchling chickens. Fertile chicken eggs were air cell injected with vehicle, perfluorooctanoic acid (PFOA)2 mg/kg, or PFOA 2 mg/kg + l-carnitine 100 mg/kg, and then incubated to ED6, 10, 15, 19, or hatch. (**A**) heart weight of ED6 chicken embryos (*N* = 10 per group); (**B**) heart weight of ED10 chicken embryos (*N* = 9–10 per group); (**C**) heart weight of ED15 chicken embryos (*N* = 9–11 per group); (**D**) liver weight of ED15 chicken embryos (*N* = 9–11 per group);(**E**) slim body weight of ED19 chicken embryos (*N* = 4–5 per group); (**F**) heart index of ED19 chicken embryos (*N* = 4–5 per group); (**G**) liver index of ED19 chicken embryos (*N* = 4–5 per group); (**H**) slim body weight of hatchling chickens (*N* = 5–8 per group); (**I**) heart index of hatchling chickens (*N* = 5–8 per group); (**J**) liver index of hatchling chickens (*N* = 5–8 per group); (**K**) hatchability of hatchling chickens (total number hatched/total number reached hatchling size) (*N* = 6–9 per group); (**L**) heart rates of hatchling chickens (*N* = 5 per group). * Statistically different from control group (*p* < 0.05). ^#^ statistically different from PFOA 2 mg/kg group (*p* < 0.05). + Specified treatment is present at the indicated group; − specified treatment is not present at the indicated group. Error bars represent standard derivation (SD).

The heart weights of ED6, 10, and 15 chicken embryos, slim body weight, heart index, and the liver index of ED19 chicken embryos and hatchling chickens, as well as the hatchability of hatchling chickens, are reported in Figure 1A–K. No statistical differences were observed among the groups.

### 2.2. Heart Rates of Hatchling Chickens

The heart rates of the hatchling chickens treated with vehicle, PFOA 2 mg/kg, or PFOA 2 mg/kg + l-carnitine 100 mg/kg were assessed with electrocardiography. Consistent with previous results, exposure to PFOA 2 mg/kg resulted in a significantly elevated heart rate relative to the control animals, while co-treatment with 100 mg/kg l-carnitine effectively reverted such changes (Figure 1L).

### 2.3. Electron Spin Resonance (ESR)Results of the Reactive Oxygen Species (ROS)Levels in ED6, 10, 15, and 19 Chicken Embryo Hearts and Hatchling Chicken Hearts

**Figure 2:**
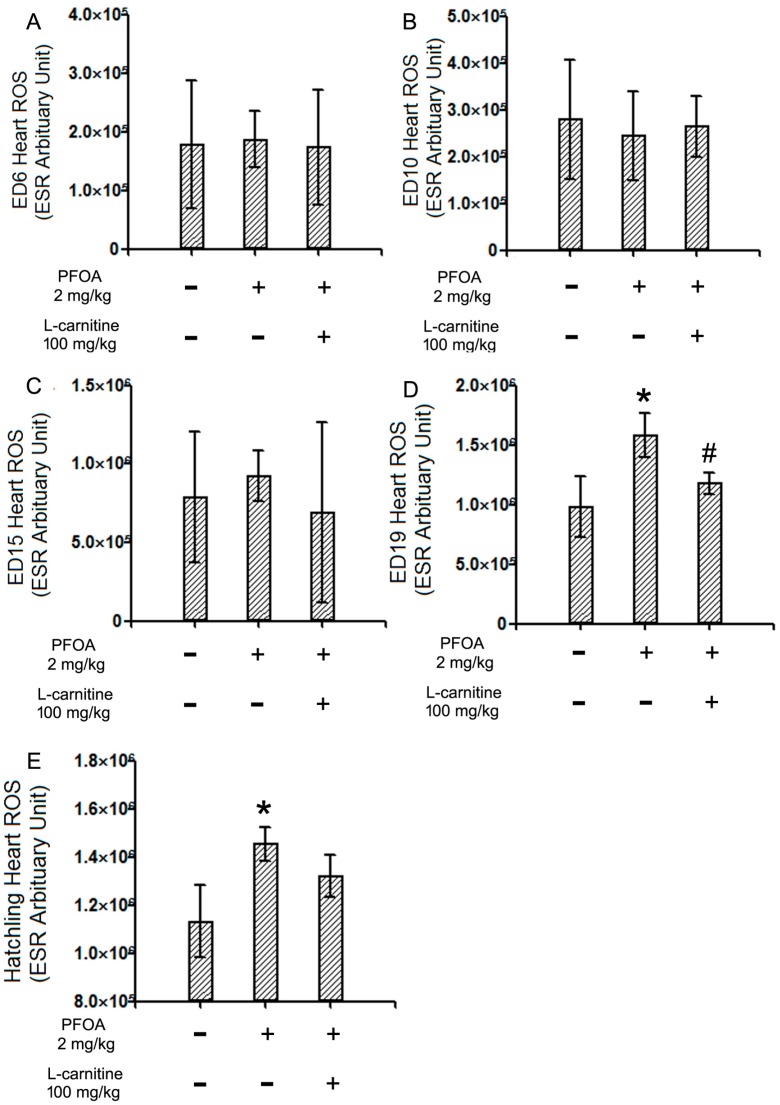
ESRresults of the reactive oxygen species (ROS) levels in ED6, 10, 15, and 19 chicken embryo hearts and hatchling chicken hearts. Fertile chicken eggs were air cell injected with vehicle, PFOA 2 mg/kg, or PFOA 2 mg/kg + l-carnitine 100 mg/kg, and then incubated to ED6, 10, 15, 19, or hatch. At desired stages, hearts were collected and subjected to an electron spin resonance (ESR) assay for ROS. The parameters of ESR were: central field 3480 G, sweep width 250 G, static field 3355 G, microwave bridge frequency 9.86 GHz, power 12.91 mW, step 2 dB. The signal for ROS was read at 3493 G. (**A**) ESR results of ED6 chicken embryo hearts; (**B**) ESR results of ED10 chicken embryo hearts; (**C**) ESR results of ED15 chicken embryo hearts; (**D**) ESR results of ED19 chicken embryo hearts; (**E**) ESR results of hatchling chicken hearts. * Statistically different from control group (*p* < 0.05). *N* = 3–4 per group. ^#^ Statistically different from PFOA 2 mg/kg group (*p* < 0.05). + Specified treatment is present at the indicated group; − specified treatment is not present at the indicated group. Error bars represent standard derivation (SD).

With the ESR method, the ROS levels of chicken embryo hearts from different stages (ED6, 10, 15, and 19), as well as hatchling chicken hearts, were assessed. The results indicated that no significant changes to ROS levels were present in ED6, 10, or 15 chicken embryo hearts (Figure 2A–C), while a significant increase in the ROS levels was detected in ED19 chicken embryo hearts and hatchling chicken hearts (Figure 2D,E). Meanwhile, l-carnitine co-treatment effectively reverted the ROS surge in ED19 chicken embryo hearts (Figure 2D). In hatching chickens, a trend of decrease was observed in the l-carnitine co-treatment group hearts compared to the PFOA treated group hearts, but this was not statistically significant (*p* = 0.10, Figure 2E).

### 2.4. ESR Results of the Nitric Oxide (NO)Levels in ED6, 10, 15, and 19 Chicken Embryo Hearts and Hatchling Chicken Hearts

**Figure 3:**
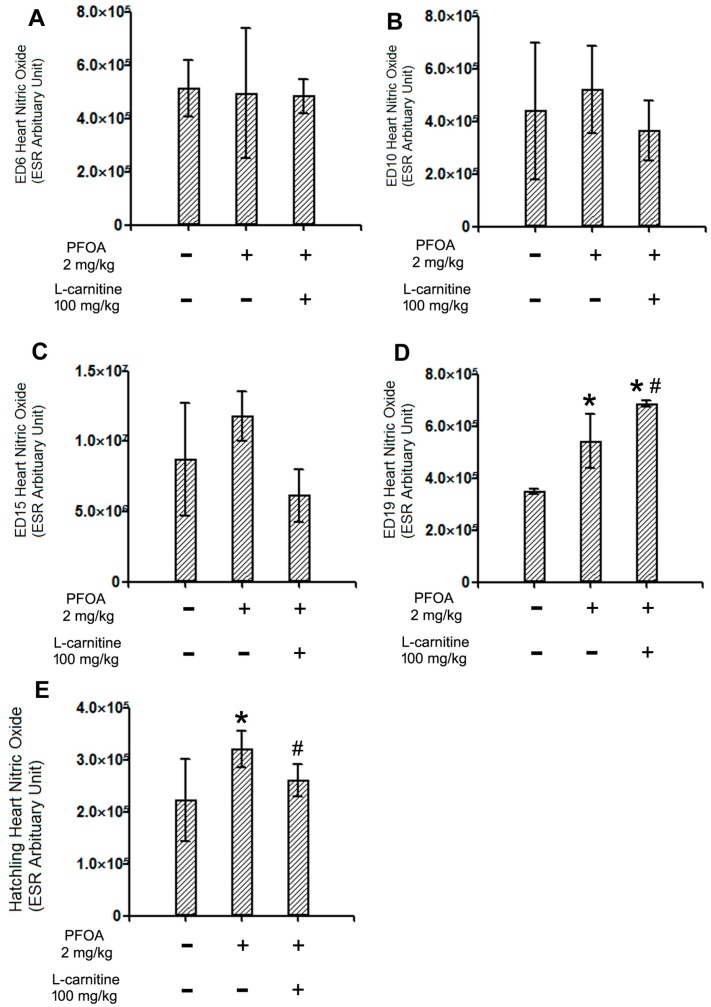
ESR results of the nitric oxide (NO) levels in ED6, 10, 15, and 19 chicken embryo hearts and hatchling chicken hearts. Fertile chicken eggs were air cell injected with vehicle, PFOA 2 mg/kg, or PFOA 2 mg/kg + l-carnitine 100 mg/kg, and then incubated to ED6, 10, 15, 19, or hatch. At desired stages, hearts were collected and subjected to an electron spin resonance (ESR) assay for NO levels. The parameters of ESR were: central field 3480 G, sweep width 250 G, static field 3355 G, microwave bridge frequency 9.86 GHz, power 12.91 mW, step 2 dB. The signal for nitric oxide was read at 3437 G. (**A**) ESR results of ED6 chicken embryo hearts; (**B**) ESR results of ED10 chicken embryo hearts; (**C**) ESR results of ED15 chicken embryo hearts; (**D**) ESR results of ED19 chicken embryo hearts; (**E**) ESR results of hatchling chicken hearts. *N* = 3–5 per group. * Statistically different from control group (*p* < 0.05). ^#^ Statistically different from PFOA 2 mg/kg group (*p* < 0.05). + Specified treatment is present at the indicated group; − specified treatment is not present at the indicated group. Error bars represent standard derivation (SD).

As described in Section 2.3, the NO levels of the chicken embryo and hatchling chicken hearts were assessed with the ESR method. Similar to the ROS results, no remarkable changes were observed in the NO levels in ED6, 10, or 15 chicken embryo hearts (Figure 3A–C). On the other hand, a significant increase in the NO level was found in the ED19 chicken embryo hearts following 2 mg/kg PFOA exposure. Moreover, co-treatment with 100 mg/kg l-carnitine significantly further increased the NO levels in ED19 hearts (Figure 3D). In the hatchling chicken hearts, however, PFOA still significantly elevated the NO levels, while l-carnitine significantly decreased the NO levels relative to PFOA-exposed animals (Figure 3E).

### 2.5. Western Blotting for p65 Translocation in ED10, 15, and 19 Chicken Embryo Hearts and Hatchling Chicken Hearts

**Figure 4:**
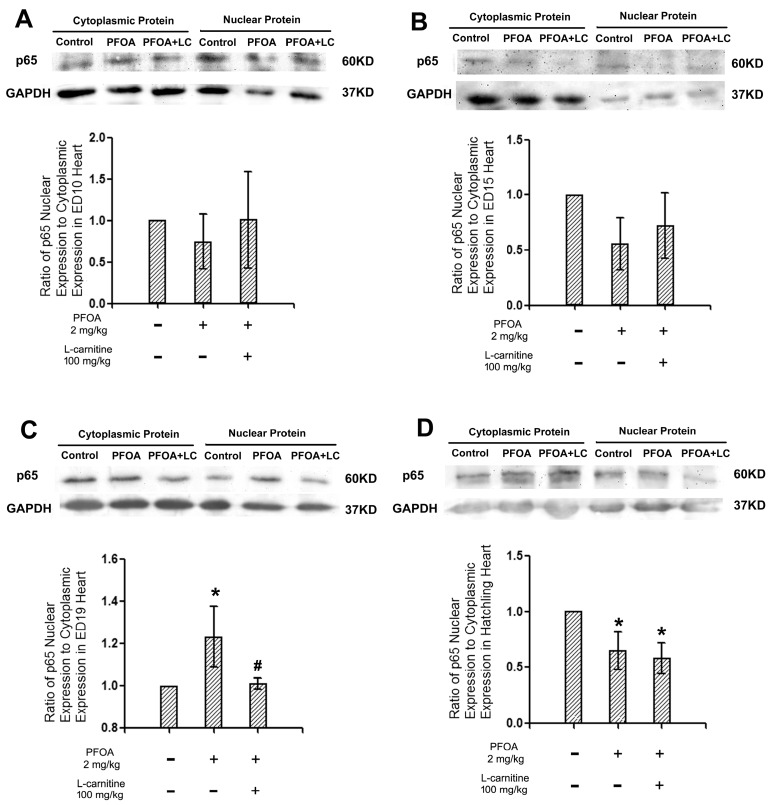
Western blotting for p65 translocation in ED10, 15, and 19 chicken embryo hearts and hatchling chicken hearts. Fertile chicken eggs were air cell injected with vehicle, PFOA 2 mg/kg, or PFOA 2 mg/kg + l-carnitine (LC) 100 mg/kg, and then incubated to ED 10, 15, 19, or hatch. At the desired stage, hearts were collected and subjected to the nuclear and cytoplasm protein extraction kit (KGP1100, KeyGenBioTECH, Nanjing, China). Cytoplasmic and nuclear protein extractions from same animals were assessed with western blotting, where the ratios of nuclear to cytoplasmic for p65 was calculated as the p65 translocation level. (**A**) p65 translocation levels of ED10 chicken embryo hearts; (**B**) p65 translocation levels of ED15 chicken embryo hearts; (**C**) p65 translocation levels of ED19 chicken embryo hearts; (**D**) p65 translocation levels of hatchling chicken hearts. *N* = 3–4 per group. * Statistically different from control group (*p* < 0.05). ^#^ Statisticallydifferent from PFOA 2 mg/kg group (*p* < 0.05). + Specified treatment is present at the indicated group; − specified treatment is not present at the indicated group. Error bars represent standard derivation (SD). GAPDH: Glyceraldehyde 3-phosphate dehydrogenase.

Western blotting was used to examine the translocation of the NF-κB p65 protein in the chicken embryo and hatching chicken hearts. Different trends in different embryonic stages were revealed. At ED10 and ED15, no statistical changes were observed among the groups (Figure 4A,B). At ED19, a significant increase of p65 translocation was observed in PFOA treated animals, while l-carnitine effectively returned the p65 translocation level back to the control level (Figure 4C). In hatchling chickens, however, a significant decrease in the p65 translocation level was observed in both PFOA treated animals and PFOA + l-carnitine treated animals (Figure 4D).

### 2.6. Western Blotting for Inducible Nitric Oxide Synthase (iNOS) in ED19 Chicken Embryo Hearts and Hatchling Chicken Hearts

**Figure 5:**
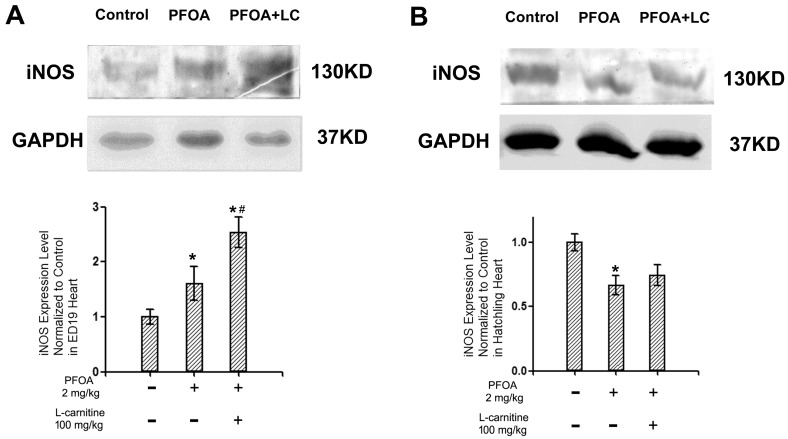
Western blotting for inducible nitric oxide synthase (iNOS) in ED19 chicken embryo hearts and hatchling chicken hearts. Fertile chicken eggs were air cell injected with vehicle, PFOA 2 mg/kg, or PFOA 2 mg/kg + l-carnitine 100 mg/kg, and then incubated to ED19 or hatch. At the desired stage, hearts were collected, protein was extracted with RIPA buffer supplemented with PMSF and then assessed with western blotting, and the expressions were normalized to control animals. (**A**) iNOS relative expression levels of ED19 chicken embryo hearts; (**B**) iNOS relative expression levels of hatchling chicken embryo hearts. *N* = 3 per group. * Statistically different from control group (*p* < 0.05). ^#^ statistically different from PFOA 2 mg/kg group (*p* < 0.05). + Specified treatment is present at the indicated group; − specified treatment is not present at the indicated group. Error bars represent standard derivation (SD).

Since significant differences were observed for the levels of NO in ED19 chicken embryos and hatchling chickens, as indicated by the ESR results, the inducible nitric oxide synthase (iNOS) levels in these animals were assessed with western blotting. As shown in Figure 5, the protein levels of iNOS significantly increased in the ED19 hearts from PFOA/l-carnitine co-treated animals, relative to both the control and PFOA treated animals (Figure 5A). On the other hand, the expression of iNOS was suppressed in PFOA treated animals relative to the control group, while l-carnitine co-treatment alleviated such changes (Figure 5B).

## 3. Discussion

In this study, the ROS and NO levels in PFOA-exposed chicken embryo hearts at different stages were evaluated with the ESR method, and ROS/NF-κB p65 and iNOS/NO were investigated as potential underlying mechanisms. The effects of l-carnitine were also investigated, whose protective effects have been reported previously [14]. In the current study, no significant differences in the ROS and NO levels were observed in the early stage embryo hearts, but our results indicated that ROS and NO might play important roles in late stage embryo hearts, which were associated with NF-κB p65 and iNOS.

### 3.1. Perfluorooctanoic Acid (PFOA)Induced Developmental Cardiotoxicity in a Chicken Embryo

It has been well-established that PFOA could induce developmental toxicity, with endpoints such as embryo loss, a low birth weight, and high post birth mortality [11,27]. No definitive explanations were found for these changes. One of the vital organs during embryo development is the heart, which starts to develop at a very early stage [28], making it vulnerable to extragenous disruptions. A chicken embryo is an established model for the study of development and various diseases, including tumor and cardiovascular diseases [29]. In previous studies, PFOA has been associated with developmental cardiotoxicity in chicken embryos, in terms of an altered morphology and function, which was achieved at an exposure level comparable to those of an occupationally exposed human [12], suggesting the human health relevancy and need for further mechanistic studies. Interestingly, the commonly-accepted mechanism of action for PFOA, PPAR α activation, failed to explain most of the observed effects [13,14]. Meanwhile, a nutrient, l-carnitine, was found to be quite effective against such changes [14], which was consistent with various reports that l-carnitine may protect the heart [17,18]. In the current study, such protection was once again confirmed, as l-carnitine co-treatment reverted the heart rate elevation induced by PFOA in hatchling chickens, thus suggesting its potential to be utilized in cardiovascular development protection.

### 3.2. l-Carnitine, PFOA, Reactive Oxygen Species (ROS), and Nuclear Factor Κ-Light Chain-Enhancer of Activated B cells (NF-κB)

Both l-carnitine and PFOA are involved in fatty acid β-oxidation, which is a process closely coupled to ROS generation [30]. It has been reported that PFOA could induce ROS in various cell types and organisms [22,31], which might contribute to its toxicity. l-carnitine has been reported to possess anti-oxidant effects in multiple studies [20,32]; however, it may also promote ROS generation since it facilitates β-oxidation [23]. In the current study, the ROS levels were determined in ED6, 10, 15, 19, and hatchling chicken hearts. While no remarkable changes were observed in earlier stage hearts, a significant increase in the ROS level was observed in ED19 chicken embryo hearts exposed to PFOA, suggesting the role of ROS in PFOA-induced developmental cardiotoxicity. This result is consistent with previous studies with PFOA [22,31]. Meanwhile, co-treatment with l-carnitine significantly decreased the ROS level in ED19 hearts, suggesting the anti-oxidant effects of l-carnitine, which may help to explain the protective effects exerted by l-carnitine. The observed effects make good sense in terms of chicken embryo development, in which ED19 is the stage with relative hypoxia [33]. Similarly, in hatchling chicken hearts, the elevated ROS levels are still present in PFOA-exposed animals, while l-carnitine co-treatment decreased the ROS level to the point that it was not significantly different from that of the control group. However, the extent of anti-oxidant effects of l-carnitine in those hatchlings seems to be somewhat decreased, as the ROS level in the co-treatment group did not statistically differ from the level in the PFOA treated group. This might be explained by the extensive labor required for the hatching process [33], in which β-oxidation serves as an important resource of energy [34]. An abundant oxygen supply post hatch might be another reason for this result.

Several signaling pathways could be activated by ROS, and one of the most important signaling pathways is the NF-κB [35]. The elevation of p65 is associated with an increased ROS level [36]. In the current study, increased p65 translocation was observed in ED19 chicken embryos, while l-carnitine returned it to the normal level, suggesting that l-carnitine exerted anti-oxidant effects in ED19 chicken embryo hearts, consequently decreasing the activation of p65, which is consistent with the ESR data of ROS levels. Interestingly, different results were observed in hatchling chicken hearts: PFOA and/or l-carnitine both suppressed p65 translocation. Notably, in ED15 embryo hearts, p65 translocation also showed a similar pattern to the hatchling hearts, although this was not statistically significant. This contradiction suggests that PFOA might be able to modulate the p65 signaling pathway by other means than ROS generation in hatchling chickens. The “normal” pattern of p65 translocation observed at ED19 might actually be a combination of ROS generation induced changes, along with the modulation by PFOA via other routes. This could help to explain the relatively small percentage change inp65 translocation in ED19 hearts. Considering the fact that PFOA is involved in immunotoxicity and tumor formation [9,10], both of which involve the NF-κB pathway, further study focusing on the interactions between PFOA and NF-κB in developing organisms is required.

### 3.3. l-Carnitine, PFOA, NO, and iNOS

Nitric oxide (NO) is an important signaling molecule in the cardiovascular system. Besides its well known effect of vasodilatation, it also has roles in inflammation and cardiovascular diseases [37]. In the developing heart, NO is known to alleviate hypoxia damage and vascular modeling [38,39], and thus, the NO levels in developing chicken embryo hearts were assessed as a potential mechanism of action for PFOA and/or l-carnitine. While no remarkable changes were detected for ED6, 10, and 15 hearts, a significant elevation of NO in ED19 in PFOA exposed hearts was revealed. Interestingly, co-treatment with l-carnitine further increased the NO levels. The elevated NO levels in PFOA-exposed hearts might represent compensation, while l-carnitine mediated a further increase in the NO level that may contribute to its protective effects. In hatchling hearts, however, such changes were no longer present; while PFOA still elevated the NO levels, co-treatment with l-carnitine exerted the reverse effects. The very different oxygen supply condition and labor required during hatching might contribute to such pheromones. To address such intriguing results, the expression levels of iNOS in ED19 and hatchling hearts were assessed. iNOS is one of the major resources of NO in living organisms, and is likely to be associated with vascular dysfunction [40]. While many existing studies correlated iNOS to negative outcomes in the cardiovascular system [40,41], our data have suggested its protective potential against developmental cardiotoxicity in ED19 chicken embryos, which seems to be a transient effect as such changes disappear after hatching, within a period of 48–72 h. In hatchling chicken hearts, iNOS expression seems to be slightly suppressed by PFOA exposure, while co-treatment with l-carnitine abolished such changes, suggesting a role of iNOS and NO in PFOA-induced developmental cardiotoxicity. Moreover, the inconsistent results of the NO and iNOS levels in PFOA treated animals suggested that other enzymes may contribute to the NO level elevation. Possible enzymes include endothelial nitric oxide synthase (eNOS), neuronal nitric oxide synthase (nNOS) [40], and xanthine oxidase [42]. The exact role of iNOS and NO in the hatching process and PFOA-induced developmental cardiotoxicity will be further pursued.

## 4. Materials and Methods

### 4.1. Materials

PFOA, l-carnitine, and the chemicals used for ESR were purchased from Sigma (St. Louis, MO, USA). Fertile chicken eggs (*Gallus gallus*) were purchased from Linwen Trading Co. Ltd. (Jining, China). The antibody against p65 was purchased from Abcam (Cambridge, MA, USA). The antibody against GAPDH was purchased from ZSbio (Beijing, China). Other chemicals and consumables were of the highest grade obtainable.

### 4.2. Chicken Embryo Treatment and Incubation

Upon arrival, fertile chicken eggs were cleaned with povidone iodide and candled in a dark room to mark the air cell with pencil. The eggs were then air cell injected with vehicle or pre-made dosing solutions to achieve the doses of PFOA 2 mg/kg or PFOA 2 mg/kg + l-carnitine 100 mg/kg. For the detailed injection method, please refer to Jiang et al. [12]. The dose of PFOA was based on the results of the hatchling chicken serum PFOA concentration [12], while the dose of l-carnitine was determined according to the reference dose set by the US Food and Drug Administration (USFDA) [43]. The doses were calculated so that a 0.1 μL/g injection volume resulted in the desired doses to the whole egg. To facilitate the homogenization of l-carnitine in the dosing suspension, 10% ethanol and 20% distilled water were included in the dosing solution. Injected eggs were initially incubated in a Keyu incubator (Dezhou, China) at 37.9 °C Celsius and 50% humidity, and the conditions were gradually changed to 37.1 °C Celsius and 70% humidity (temperature and humidity were controlled automatically by the incubator). Eggs were turned every 3 h until ED19. Hatched chickens were kept in a warmed brood box with water provided until the heart function assessment and sample collection (within 24 h post hatch). All the procedures for the handling of chicken embryos and hatchling chickens were approved by the Institutional Animal Care and Use Committee (IACUC) of Qingdao University (Approved protocol 2015018, 5 March 2015).

### 4.3. Chicken Embryo Sample Collection

At the desired time points, the eggs were opened up, the embryos were carefully removed from the eggs and quickly decapitated, and the hearts were carefully dissected out and immediately frozen in a −80 °C freezer until use.

### 4.4. Hatchling Chicken Heart Rate Assessment and Sample Collection

Hatchling chickens (approximately 12–24 h post hatch) were anesthetized with 33 mg/kg pentobarbital via an intraperitoneal injection and the heart rate was assessed with BL-420 E+ (Taimeng, China), as described in Jiang et al. [14], before being quickly decapitated while still under anesthesia, at which point the hearts were collected and frozen in a −80 °C freezer until further use.

### 4.5. ESR

ED6, 10, 15, 19, and D1 hatchling chicken hearts were assessed with ESR for reactive oxygen species (ROS) and nitric oxide (NO). Briefly, the tissues were homogenized with 5× volume ESR buffer (10 mM *N*-tert-butyl-a phenylnitrone, 2 mM diethylene-triaminepentacetic acid, 0.1 mM ethylene diaminetetraacetic acid, 10 mM 4-(2-hydroxyethyl)-1-piperazineethanesulfonic acid in phosphate buffered saline, pH 7.4) and centrifuged at 12,000 rpm for 20 min at 4 °C Celsius. The resulting supernatant was isolated and mixed with 10 μL each of 0.5 M Sodium dithionite, 10 mM L-argentine, 0.3 M ferrous sulfate, 10 mM calcium chloride, and 0.6 M sodium diethyldithiocarbamate. Samples were then incubated in dark at 37 °C Celsius for 1 h, and were then put on ice and mixed with 200 μL ethyl acetate thoroughly. The samples were then centrifuged at 1100 rpm for 10 min, and 140 μL of ethyl acetate phase was used for ESR detection. The ESR parameters used were: central field: 3480 G, sweep width: 250 G, static field: 3355 G. Microwave bridge frequency: 9.86 GHz, power 12.91 mW, step 2 dB. The signal for nitric oxide was read at 3437 G, while the signal for ROS was read at 3493 G.

### 4.6. Western Blotting

ED10, 15, 19, and D1 hatchling chicken hearts were investigated with western blotting for the translocation status of NF-κB p65 and the protein expression levels of iNOS. For p65, the cytoplasmic and nuclear protein extractions were prepared with the nuclear and cytoplasm protein extraction kit (KGP1100, KeyGenBioTECH, Nanjing, China) following the manufacturer provided protocol. For iNOS, independent samples were extracted with a radio immunoprecipitation assay buffer (RIPA) supplemented with 1:100 PMSF (Beyotime, Beijing, China). Western blotting was performed as described in Wang et al. [42], with slight modifications. P65 antibody (ab3523, dilution 1:2000) was purchased from Abcam (Cambridge, UK). iNOS antibody (bs-2072R, dilution 1:1000) was purchased from Bioss (Beijing, China). GADPH antibody (TA-09, dilution 1:3000) was purchased from Nobleryder (Beijing, China). Blots were visualized with an enhanced chemiluminescence kit (Millipore, Billerica, MA, USA), along with a UVP 810 gel imager (UVP, Upland, CA, USA), and then analyzed with ImageJ (NIH, Bethesda, MD, USA). The results were normalized to the internal control (GAPDH) of the corresponding samples. For the p65 translocation status assay, the ratio of nuclear p65 to cytoplasmic p65 from the same animal was calculated, and semi-quantified as a ratio to the control sample on the same gel (the cytoplasmic extraction and nuclear extraction of one animal were always assessed on the same gel). Three independent samples were used in each group.

### 4.7. Statistical Analysis

Statistical analysis was performed with SPSS 17.0 (SPSS Inc. Chicago, IL, USA). One-way analysis of variance (ANOVA) was performed to detect differences. When ANOVA returned significant results, post-hoc least significant difference (LSD) tests were used to compare the results among groups. The results were considered statistically significant when *p* < 0.05.

## Supplementary Materials

Supplementary File 1

## Abbreviations

NF-κB	Nuclearfactor κ-light chain-enhancer of activated B cells
PFOA	perfluorooctanoic acid
ESR	electron spin resonance
ROS	reactive oxygen species
NO	nitric oxide
iNOS	inducible nitric oxide synthase
ED	Embryonic day
